# Hyperdominant left anterior descending artery continuing across left ventricular apex as posterior descending artery coexistent with aortic stenosis

**DOI:** 10.1186/1749-8090-2-42

**Published:** 2007-10-21

**Authors:** Kalyana Javangula, Pankaj Kaul

**Affiliations:** 1 Department of Cardiothoracic Surgery, Yorkshire Heart Centre, Leeds General Infirmary, Great George Street, Leeds, LS1 3EX UK; 2 Yorkshire Heart Centre, Leeds General Infirmary, Great George Street, Leeds, LS1 3EX UK

## Abstract

We describe, in a 61 year old man, with coexistent aortic stenosis, the anomalous origin of posterior descending artery (PDA) from a stenotic left anterior descending (LAD) artery, as its continuation across the left ventricular apex, in the presence of a normally arising and atretic proximal right coronary artery. The patient underwent mechanical aortic valve replacement and triple coronary artery bypass grafting and made an uneventful recovery. To the best of our knowledge, origin of PDA as a continuation of LAD across the left ventricular apex in the presence of a normally arising but atretic proximal right coronary artery has never been described in literature before. There is one previous case report of continuation of LAD as PDA across the left ventricular apex in a patient with single left coronary coronary artery with an absent right coronary ostium. As the blood supply to the entire interventricular septum is derived from this "hyperdominant" LAD system, stenosis of LAD can be catastrophic. A review of literature of the anomalies of right coronary artery and, in particular, of its anomalous origin from LAD and its coexistence with aortic stenosis, is presented.

## Case presentation

**Figure 1:**
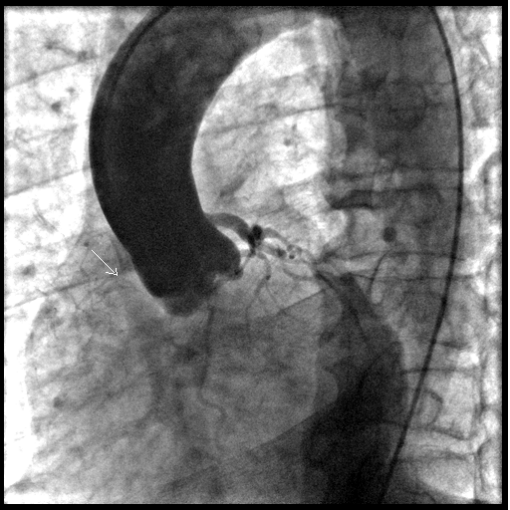
Aortic root angiogram showing normally situated left and right coronary ostia, normal left main stem and small, atretic right coronary artery.

**Figure 2:**
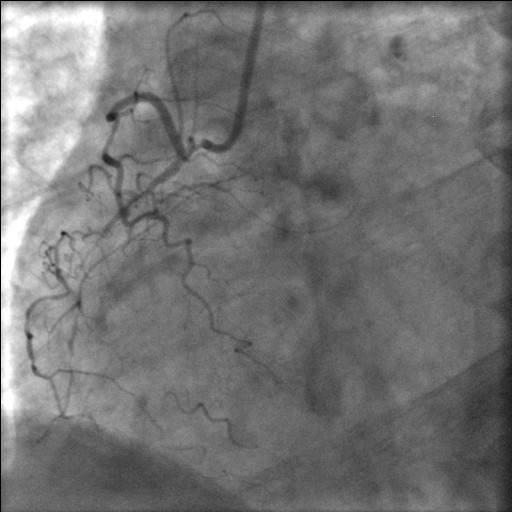
Selective right coronary angiogram showing a normally situated coronary ostium, a small atretic RCA giving off SA nodal, right atrial and right ventricular branches and petering out thereafter.

**Figure 3:**
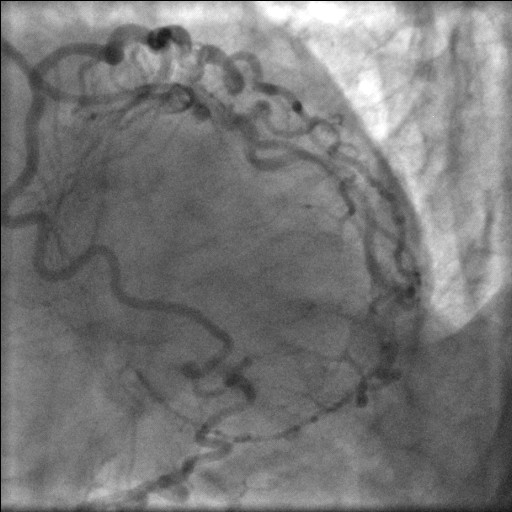
Selective left coronary angiogram demonstrating normal left main stem and circumflex and continuation of LAD as PDA.

**Figure 4:**
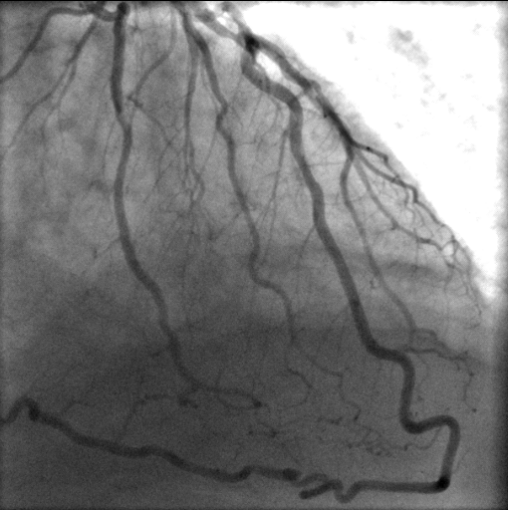
Bifurcation stenosis of LAD and Dx and continuation of LAD as PDA.

**Figure 5:**
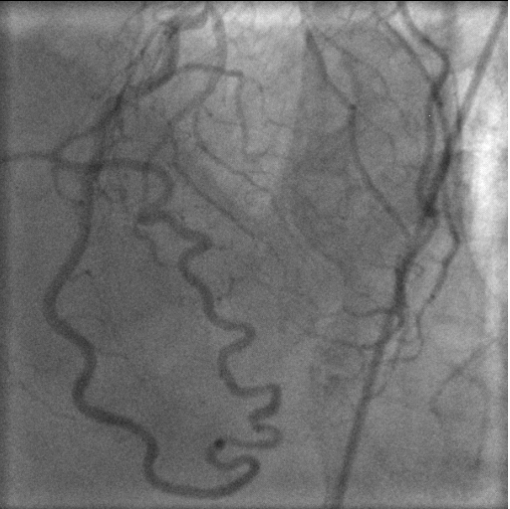
LAD continues as PDA and then as RCA rightward of the crux, where it gives off the left ventricular branch to the inferior surface of the left ventricle.

**Figure 6:**
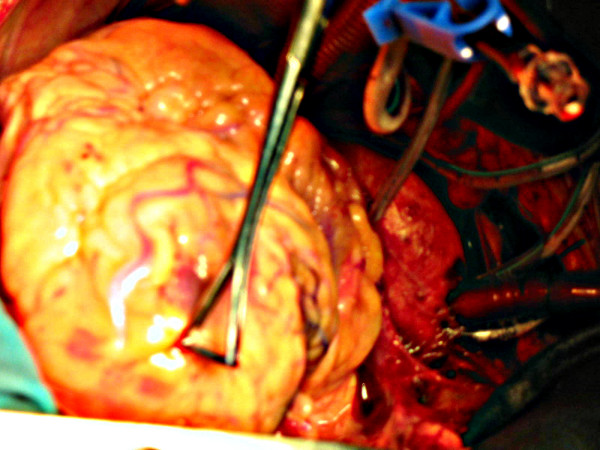
LAD continues across the LV apex as PDA.

A 61 year old current male smoker presented with moderate exertional angina and shortness of breath. Significant comorbidities included intermittent claudication, chronic bronchitis and asbestosis related benign pleural disease. Examination revealed a harsh ejection systolic murmur across the whole precordium, radiating to carotid area. Echocardiogram confirmed moderate aortic stenosis with a peak gradient of 62 mm Hg and mean gradient of 34 mm Hg across the aortic valve, mild aortic regurgitation, left ventricular hypertrophy and preserved left ventricular function. Left heart catheterization demonstrated a gradient of 30 mm Hg across the aortic valve. Aortic root angiogram showed mild aortic regurgitation and a small and atretic normally arising proximal right coronary artery and a normally arising left coronary artery (Fig 1). The selective right coronary angiogram demonstrated the atretic right coronary artery (RCA) supplying the SA nodal, right atrial and the proximal right ventricular branches and petering out thereafter (Fig 2). The selective left coronary angiogram showed a normal left main stem (Fig 3), ostial and mid vessel stenotic disease in left anterior descending artery (LAD) and a normal circumflex artery (Fig 4). LAD continued across the left ventricular apex as posterior descending artery (PDA), running along the posterior interventricular septum up to the atrioventricular groove, where it gave off the left ventricular branch to the inferior surface of left ventricle and thereafter continued as the distal RCA without establishing any communication with the atretic proximal RCA (Fig 5). Left ventriculogram confirmed well preserved left ventricular function. At operation, there was moderately severe left ventricular hypertrophy. The left anterior descending artery, after running its normal course in the anterior interventricular groove, ran across the left ventricular apex (Fig 6) to gain the posterior interventricular groove, where it continued as the posterior descending artery up to the crux of the heart, thereafter ascending for a brief distance as the distal right coronary artery after having given off a smaller left ventricular branch to the inferior surface of the left ventricle. There was significant palpable disease in proximal and mid LAD as well as in its large diagonal (Dx) branch. There was no continuity between the atretic proximal RCA and the anomalous distal RCA. Circumflex artery was a normal sized vessel with a normal sized obtuse marginal branch. Aortic valve was tricuspid in configuration, moderately stenotic with fused commissures and thickened and calcified leaflets.

Employing cardiopulmonary bypass with moderate hypothermia and right superior pulmonary vein vent and using both antegrade and retrograde cold blood cardioplegic arrest, aortic valve replacement with 21 mm St Jude mechanical prosthesis and triple coronary artery saphenous vein bypass grafting to LAD, Dx and PDA was performed. Patient made uncomplicated postoperative recovery, was transferred to ward on 2^nd ^postoperative day and home on 8^th ^postoperative day.

## Discussion

No two coronary anatomic patterns are alike and there is a wide range of variability within the normal distribution. Coronary artery anomalies represent marked deviations from normal but, fortunately, have a relatively constant incidence of less than 1.5% across different racial groups [1-6]. Anatomically, the anomalies can be divided into those of origin and distribution and those with fistulae. Clinically, the anomalies are arbitrarily divided into benign and malignant, based on their potential to cause myocardial ischaemia. Of the benign anomalies, the three most common are 1) separate origins of LAD and CX in left sinus of Valsalva 2) origin of CX from right sinus of Valsalva or from right coronary artery 3) ectopic origin of right coronary artery from aorta, a high origin being particularly prone to accidental cross clamping or side clamping, or transection during aortotomy [1]. Of the malignant abnormalities, the most common anomaly, by a wide margin, is the ectopic origin of right coronary artery from the left sinus of Valsalva [1].

Right coronary artery anomalies, in turn, can involve the origin and distribution of the vessel. The most common and the most significant of these is the ectopic origin of right coronary artery from the left sinus of Valsalva. The anomalous right coronary artery, which is difficult to cannulate, arises from an ectopic ostium located anterior to left main ostium in the left sinus of Valsalva and passes anteriorly between aorta and pulmonary artery before reaching the AV groove. Ostial compression during ventricular diastole or aortic distension during exercise may result in myocardial ischaemia and the clinical sequelae, though rare, may result in angina, myocardial infarction, ventricular tachycardia, syncope or sudden death in the absence of coronary atherosclerosis [7-12]. Rarely, the ectopic RCA orifice may be located posterior to the left main ostium when the right coronary artery pursues a course posterior to the aorta where it is free from great vessel compression. Out of 136 subjects with ectopic origin of right coronary artery from left sinus of Valsalva, Yamanaka et al found 135 with ectopic orifice anterior to left main ostium and only one with a posterior orifice [1].

Ectopic origin of RCA from posterior sinus of Valsalva, by contrast, is an extremely rare anomaly which runs a benign course with the right coronary artery having a normal distribution. It is discovered accidentally and is not associated with any ischaemic symptoms [13].

An abnormally high take off of right coronary artery from above the sinotubular line with a normal distribution [14,15] or an abnormal initial intraortic course [16] with implications regarding angiography, angioplasty [17] or surgery has been described. Right coronary artery has been described at least once to arise below the aortic valve [18]. None of the above, however, is associated with malignant compression.

Rarely, the atrioventricular branch of right coronary artery is contiguous with the main trunk of circumflex artery, and the distal LAD with distal PDA, with bi-directional blood flow and an open-ended circulation. This anomaly is benign and may serve as a collateral source should an obstruction develop at either end and should not be misinterpreted as a functioning collateral with occlusion of a proximal coronary artery [19].

Double right coronary arteries have been described. Both might develop obstructive atherosclerotic disease [20] and the two arteries might give anatomically diverse branches [21].

Cheatham described the origin of right coronary artery from descending thoracic aorta in an infant with hypoplastic left heart syndrome at autopsy [22]. Radke [23], Yao [24] and Lessick [25] separately reported the anomalous origin of right coronary artery from main pulmonary artery, the ischaemic potential of the anomaly and the desirability of reconstitution of double ostium coronary system.

The single left coronary artery is further classified into L1 and L2 groups by the Lipton scheme [26]. In L1 pattern, the right coronary artery is congenitally absent, the CX is markedly dominant and provides the posterior descending branch and thereafter ascends in the right AV groove where it provides branches to right atrium and right ventricle. Yamanaka reported 20 such anomalies in 126,595 diagnostic angiograms and 1,686 coronary anomalies (0.016% incidence and 1.2% of all anomalies) and remarked on the generally benign course of this anomaly [1]. In the even rarer L2 subtype, the right coronary ostium is congenitally absent and the right coronary artery arises from left main stem or from the proximal branches of left coronary artery. If arising from the main stem, the anomalous right coronary artery can course anterior [1], posterior [27] or in between [28] the great vessels, the last anomaly being not only the most common but also most likely to be associated with external compression and ischaemic symptoms.

Only 10 cases have been described of the origin of right coronary artery from LAD in L2 subtype of the single left coronary artery [29-35]. In most of these, the right coronary artery arises from proximal or mid LAD and courses to the right to gain the right AV groove or acute margin of right ventricle, with the ischemic symptoms resulting from obstructive disease in LAD or rarely in RCA [29-33] although a meandering intraseptal course can sometimes cause inferior ischaemia [34]. Hamodraka et al reported an extremely unusual case of LAD continuing as posterior descending artery in posterior interventricular groove after going around the left ventricular apex in the absence of right coronary ostium – an intriguing variation on the L2 Lipton subtype, where RCA, instead of arising from proximal branches of left coronary artery arises as a distal continuation of LAD [35]. Kaul et al described a single left coronary artery with separate and composite origins of proximal and distal right coronary arteries from left anterior descending and circumflex arteries [36].

RCA has been anecdotally reported to arise from LAD in the presence of a normally situated right coronary ostium. Kamran et al reported, in a patient requiring mitral valve replacement, the incidental discovery of an anomalous right coronary artery arising from mid LAD, which coursed along the free wall of right ventricle into the right AV groove, and continued as posterior descending artery. This was associated with a separate small proximal RCA originating from right coronary sinus, with right conus, right atrial and right ventricular branches [37]. John described, in association with a normally arising small non dominant proximal RCA, an aberrant artery which originated proximal to an LAD stenosis and meandered anterior to the root of pulmonary artery and right ventricle, down the acute margin of the heart, on to the inferior surface of heart to terminate as the posterior descending artery, the patient undergoing LIMA graft to LAD [38].

Separate origin of LAD and CX from the left coronary sinus (absent left main trunk) is found with increased incidence in aortic valve disease [39]. Misawa et al described various strategies to deal with anomalous right coronary arteries during aortic valve surgery such as rotation of a free style bioprosthesis by a subcoronary technique, interposition saphenous vein bypass graft between ascending aorta and the separated artery and a subannular aortic valve to avoid obstucting the anomalous orifice [40]. Ichikawa et al described aortic valve replacement for bicuspid aortic valve stenosis with single left coronary artery [41].

Stenosis of a hyperdominant LAD which is the sole, as was the case in our patient, or predominant blood supply to the interventricular septum may have catastrophic complications. Kaul, however, described repeated successful surgical rescues of early and delayed ruptures of ventricular septum, right ventricle and aneurysmal left ventricle following massive biventricular infarction subsequent to the occlusion of a hyperdominant LAD, albeit in the presence of a normally arising but modestly distributed RCA [42].

Our patient, to the best of our knowledge, is the first patient in world literature with LAD continuing as PDA across the left ventricular apex in the presence of a normally situated right coronary ostium, although with an atretic small right coronary artery. LAD continuing as PDA across the left ventricular apex has been described once but only as a variation of single left coronary artery, in the absence of a right coronary ostium [35]. RCA has been reported to arise from mid LAD in the presence of a normally situated right coronary ostium giving rise to a small proximal right coronary, but the course has been across the free wall of right ventricle into the right AV groove [37] or across the root of pulmonary artery and the acute margin of heart [38] rather than across the LV apex as in our patient. The presence of coexistent aortic stenosis in our patient, we think, was an incidental finding rather than a component of a well known syndrome, although absent left main trunk [39], anomalous high origin of RCA [40] and single left coronary artery [41] have been described with aortic stenosis.

## Competing interests

The author(s) declare that they have no competing interests.
